# Genetic variation in patent foramen ovale: a case-control genome-wide association study

**DOI:** 10.3389/fgene.2024.1523304

**Published:** 2025-01-13

**Authors:** Bosi Dong, Yajiao Li, Fandi Ai, Jia Geng, Ting Tang, Wan Peng, Yusha Tang, Hui Wang, Zixuan Tian, Fengxiao Bu, Lei Chen

**Affiliations:** ^1^ Department of Neurology, West China Hospital of Sichuan University, Chengdu, China; ^2^ Department of Cardiology, West China Hospital of Sichuan University, Chengdu, China; ^3^ Institute of Rare Diseases, West China Hospital of Sichuan University, Chengdu, China

**Keywords:** patent foramen ovale, genome-wide association study, heart development, common variants, single-cell sequencing

## Abstract

**Background:**

Patent foramen ovale (PFO) is a congenital defect between the atria, resulting in abnormal hemodynamics. We conducted a genome-wide association study (GWAS) to identify common genetic variants associated with PFO.

**Methods:**

We performed a whole genome sequencing in a discovery cohort of 3,227 unrelated Chinese participants screened for PFO via contrast transthoracic echocardiography (cTTE). Single-nucleotide polymorphisms (SNPs) associated with PFO were further validated by Sanger sequencing and subsequently were evaluated in a validation cohort. Expression quantitative trait loci (eQTL) analysis was conducted using the GTEx database. Single-cell sequencing analyses with pseudotime trajectory modeling were employed to evaluate their expression in human fetal hearts.

**Results:**

The case-control GWAS of discovery cohort ultimately included 517 cases and 517 demographically matched controls. Of the 7,040,407 variants assessed, we identified rs1227675732 (OR = 2.903; 95% CI, 1.961 to 4.297; *p* = 3.05 × 10^−8^), rs62206790 (OR = 2.780; 95% CI, 1.864 to 4.146; *p* = 2.02 × 10^−7^), rs879176184 (OR = 2.724; 95% CI, 1.822 to 4.073; *p* = 4.30 × 10^−7^) and rs13115019 (OR = 2.437; 95% CI, 1.702 to 3.488; *p* = 5.80 × 10^−7^) as high-risk variants for PFO, while rs57922961 (OR = 0.5081; 95% CI, 0.388 to 0.666; *p* = 6.82 × 10^−7^) was identified as protective variant. These variations were replicated in the validation cohort (111 cases and 152 controls). Single-cell sequencing showed that *CNOT2*, *KCNMB4*, *MLLT10*, *IGBP1*, and *FRG1* were highly expressed with significant changes during heart development.

**Conclusion:**

The identification of susceptible loci for PFO might provide insights into the pathogenesis of PFO and contribute to understanding heart development.

**Clinical Trial Registration:**

https://www.chictr.org.cn/showproj.html?proj=40590, identifier ChiCTR1900024623.

## 1 Introduction

Patent foramen ovale (PFO) is a common congenital heart defect characterized by a potential space or separation between the primum septum and secundum septum (Snijder et al., 2016). The foramen ovale should spontaneously close with the increase in left atrial pressure and decrease in pulmonary resistance at birth, followed by fibrous adhesion of the primum and secundum septa during the first year of life (Silvestry et al., 2015). However, it fails to fuse completely in some individuals even after 3 years old, resulting in a PFO (Hagen et al., 1984). The incidence of PFO is approximately 20% in the general population and is considered a normal anatomic variant (Kutty et al., 2012). Nonetheless, PFO is associated with many neurological disorders, including cryptogenic stroke, migraine, obstructive sleep apnea, and decompression sickness (Abdelfattah et al., 2022; He et al., 2018; Shaikh et al., 2013). Additionally, PFO can exacerbate hypoxemia in patients with underlying pulmonary disorders, such as chronic obstructive pulmonary disease and pulmonary hypertension (Mojadidi et al., 2019). The prothrombotic state caused by PFO or the clot formed *in situ* within the PFO may also provoke thromboembolism and even trigger atrial arrhythmias (Ioannidis and Mitsias, 2020). Preventive measures of paradoxical flow induced by large PFO, such as transcatheter PFO closure, have proven effective in decreasing embolism recurrence in cryptogenic stroke or improving hypoxia in some states (Chen et al., 2018; Silver et al., 2007).

Despite these, PFO has not received sufficient attention. Nevertheless, with increased clinical experience, it was found a higher prevalence of PFO in siblings of patients with PFO than those without PFO (61.5% vs. 30.6%) (Arquizan et al., 2001). Family studies found that the pattern of PFO occurrence was consistent with dominant inheritance, and observations in twins suggested that inheritance was more significant than the environment (Angeli et al., 2001; Wilmshurst et al., 2004). Pedigree studies have also indicated familial clustering of PFO, particularly PFOs with large atrial shunts (Posch et al., 2010). Several physicians recommended detecting PFO in asymptomatic relatives of stroke patients to counsel them about stroke prevention or activities such as scuba diving (Arquizan et al., 2001; Takafuji et al., 2024). Therefore, it is crucial to understand the underlying genetic mechanism of PFO for improving clinical management strategies.

Clinical studies have suggested several potential genetic components that may play a role in the development of PFO. The evaluation of specific sequences of *PITX2*, *GATA4*, *TBX20*, and *NKX2-5* showed their links to PFO in patients with stroke, although subsequent studies lacked significant and stable correlations (Paolucci et al., 2021). Besides, these genes are not specific to PFO and are more responsible for severe congenital heart diseases, such as atrial septal defect (Garg et al., 2003; Lahm et al., 2021). Animal experiments also showed that embryos with incorrect expression of these genes were often nonviable (Du M et al., 2016; Gao et al., 2019). PFO mouse model studies hinted for another eight genes (*Cybrd1*, *Dst*, *Fxn*, *Lrp2*, *Mcam*, *Pgbd1*, *Sik3*, *Smad6*) related to the development of PFO (Moradi et al., 2023), while significant species-specific differences exist in heart development between humans and rodents (Cui et al., 2019). To date, no large-scale genetic studies of PFO have been conducted. In this study, we performed a genome-wide association study (GWAS) in a cohort of individuals tested for PFO to better delineate the contribution of genetic variations to the risk of this trait and their impact on cardiac development.

## 2 Materials and methods

### 2.1 Study design and participants

This two-stage case-control genome-wide association study (GWAS) involved unrelated Chinese participants recruited from both West China Hospital (hospital-based population cohort) and affiliated community clinics (natural population cohort) through advertisements, word of mouth, and referrals from clinicians or other researchers. Advertisements offered the opportunity for PFO screening at no cost and did not indicate monetary compensation. There was no specified other purpose or population selection for study participants in order to maximize subject recruitment. Participants were initially referred to the Department of Cardiology at West China Hospital for clinical interviews and PFO screening. Baseline characteristics, including age, gender, ethnicity, educational level, body mass index (BMI), smoking, alcohol consumption, regular coffee intake, and medical history, were recorded during the clinical interview. Inclusion criteria were: 1) aged 4–80 years; 2) understand and cooperate with contrast transthoracic echocardiography (cTTE). Exclusion criteria were: 1) self-reported history of severe organic diseases, or tumor; 2) non-Han ethnicity to minimize population stratification; 3) presence of any heart disease other than PFO.

There were 581 participants with PFO and 2,646 participants without PFO recruited in discovery cohort between 10 March 2020 and 18 January 2023, and 2,924 samples passed filtering (see later). Due to imbalances in baseline characteristics between cases and controls in the discovery cohort (Table 1), propensity score matching (PSM) was performed to avoid bias caused by imbalanced sample number and baseline characteristics. Propensity scores were calculated using covariates including age, gender, educational level, BMI, smoking, alcohol consumption, and coffee intake. Cases and controls were matched 1:1 using the nearest-neighbor method via the MatchIt package. Finally, 517 cases and 517 controls were used in the first discovery stage. There were 263 individuals recruited in discovery cohort between 7 February 2023 and 19 March 2024, and no samples with more than 10% missing genotypes or gender nonconformity.

**TABLE 1:** Characteristics of participants with or without PFO before and after PSM.

	Before PSM			After PSM		
	With PFO (n = 517)	Without PFO (n = 2,407)	*p*	With PFO (n = 517)	Without PFO (n = 517)	*p*
Age, mean (SD)	36.18 (14.05)	49.49 (11.17)	**<0.001** ^b^	36.18 (14.05)	36.49 (13.95)	0.721^b^
Gender, female (%)	358 (69.2)	1743 (72.4)	0.162^a^	358 (69.2)	364 (70.4)	0.735^a^
BMI, mean (SD)	22.07 (3.41)	23.93 (3.36)	**<0.001** ^b^	22.07 (3.41)	22.38 (3.38)	0.146^b^
Smoker, n (%)	69 (13.3)	395 (16.4)	0.096^a^	69 (13.3)	61 (11.8)	0.511^a^
Alcohol drinker, n (%)	68 (13.2)	677 (28.1)	**<0.001** ^a^	68 (13.2)	67 (13.0)	1.000^a^
Coffee drinker, n (%)	49 (9.5)	216 (9.0)	0.781^a^	49 (9.5)	54 (10.4)	0.678^a^
Education, n (%) <6 years 6–9 years 10–12 years >12 years	62 (12.0) 152 (29.4) 154 (29.8) 149 (28.8)	682 (28.3) 1,069 (44.4) 401 (16.7) 255 (10.6)	**<0.001** ^a^	62 (12.0) 152 (29.4) 154 (29.8) 149 (28.8)	61 (12.0) 162 (31.3) 136 (26.3) 158 (30.6)	0.635^a^

Bold indicates significant differences at p < 0.05.

^a^ χ2 test.

^b^ Student’s t test.

Written informed consent was obtained from all participants, and the study was approved by the Ethics Committee of West China Hospital of Sichuan University (No. 2020145) and registered at the Chinese Clinical Trial Register (ChiCTR1900024623) in accordance with Helsinki Declarations.

### 2.2 PFO screening

Transthoracic echocardiography (TTE) was initially performed using 1–5 MHz or 3–8 MHz multiplane transducers and a Philips IE33 ultrasound device to detect any other cardiac diseases. After TTE showed no structural or functional abnormalities, cTTE was performed. Specifically, a contrast medium containing microbubbles (an agitated solution generated by 1 mL of blood, 1 mL of air, and 8 mL of saline) was injected into the antecubital veins (Silvestry et al., 2015). Participants were assessed at rest, during a Valsalva maneuver (expiratory pressure at 60 mm Hg measured by manometer), and while coughing. As described in previous studies, enough Valsalva maneuver could enhance PFO detection (Zhao et al., 2019). Following Chinese expert consensus on the diagnosis of PFO(Chinese Consensus Expert Group On The Clinical Application Of Transesophageal Echocardiography, 2022), we measured the expiratory pressure during the Valsalva test by a standard manometer. Patients were asked to exhale into a mouthpiece connected with the manometer trying to achieve 60 mmHg and maintain the expiratory pressure for 15 s. Then patients were instructed to release on command after arrival of contrast in the right atrium. Subjects were allowed to practice the Valsalva maneuver prior to the formal test. PFO was diagnosed if three or more detected microbubbles per frame appeared in the left heart within three cardiac cycles (Maffe et al., 2010).

For the size of the PFO was quantified based on the number of detected microbubbles per frame in the left heart: Grade 1, less than 10 microbubbles; Grade 2, 10–30 microbubbles; Grade 3, more than 30 microbubbles (Maffe et al., 2010).

### 2.3 DNA preparation and whole-genome sequencing

Peripheral blood samples (5 mL) were collected from the basilic vein into standard EDTA-containing collection tube. DNA was extracted from whole blood using a MagPure Blood DNA LQ kit with a KingFisher Flex automated nucleic acid purification system (Thermo Fisher Scientific, Waltham, MA, United States) following the manufacturer’s instructions. A total of 500–1,000 ng of DNA per sample was used for DNA preparation. The integrity of gDNA was determined through gel electrophoresis. DNA were fragmented on Covaris LE220-Plus. PCR products were purified using VAHTS™ DNA Clean Beads (Vazyme Biotech, Nanjing, China) in a Tecan Evo instrument. Quality control was performed using the Agilent Bioanalyzer 4200 (Agilent Technologies, Santa Clara, CA, United States) to ensure an average fragment size of 460 bp. DNA concentrations were determined with a Tecan Infinite 200 PRO and were required to be more than 5 ng/μL. DNB preps of clinical samples generated in Tecan 480 were sequenced on the ultra-high-throughput DNBSEQ-T7 platform (MGI, Shenzhen, China).

Whole-genome sequencing reads were mapped to the human reference genome (GRCh38/hg38). The GATK best practice pipeline was applied to call variants and indels (McKenna et al., 2010). The PLINK program (v 1.9), KING (v 2.3.0) and R statistics (v 4.1.0) were used for quality control procedures. Samples were removed if they exhibited: (1) gender inconsistencies (n = 5 in cases, n = 22 in controls), (2) genotype call rates less than 90% or outlying heterozygosity (n = 41 in cases, n = 55 in controls), or (3) third-degree relative or closer relationships (n = 18 in cases, n = 162 in controls). SNPs were filtered if: (1) the call rate was less than 90%, (2) the minor allele frequency (MAF) was less than 1%, or (3) significant deviation from Hardy-Weinberg equilibrium (*p* < 10^−4^). Variants were annotated using the ensemble variant effect predictor (VEP) (McLaren et al., 2016). All annotation processes were conducted based on GRCh38 genome coordinates.

### 2.4 Sanger sequencing

Amplification reactions were performed in a total volume of 15 μL, containing 2 μL 10 × buffer (Roche), 1 μL dNTP, 0.5 μL forward primer (10 μM), 0.5 μL reverse primer (10 μM), 0.15 μL Taq DNA polymerase (Roche), 1 μL genomic DNA and 9.85 μL pure water. PCR conditions were: 2 min at 95°C, 30 cycles of 30 s at 95°C, 30 s at 65°C, and 30 s at 72°C, with a final extension for 2 min at 72°C. Alternatively, PCR was performed using PrimeSTAR Max DNA Polymerase (Takara) with polymerase activation at 98°C for 30 s, followed by 35 cycles at 98°C for 10 s and 67°C for 60 s. Primers for mutation confirmation with Sanger sequencing are shown in the Supplementary Materials. PCR fragments were purified from agarose gels, and sequencing was performed using the BigDye Terminator cycle sequencing kit (Applied Biosystems). DNA fragment analysis was performed on an ABI 3730 Genetic Analyzer (Applied Biosystems) according to the manufacturer’s instructions. Sequencing profiles shown in this manuscript were generated by SnapGene Viewer 4.0 (Chicago, IL, United States).

### 2.5 Single-cell data processing

Two public datasets from articles by Asp et al. (2019) and Cui et al. (2019) containing accessible single-cell data of human fetal hearts were included. For each dataset, gene expression UMI count values were log-normalized using Seurat v4 NormalizeData with a scale factor of 10,000. After centering and scaling with Seurat ScaleData, principal component analysis (PCA) was performed on variable genes with Seurat RunPCA and embedded in two-dimensional Uniform Manifold Approximation and Projection (UMAP) plots with Seurat RunUMAP on 10 (data from Asp et al. (2019)) or 30 (data from Cui et al. (2019)) principal components. Clusters showing expression levels of *CNOT2*, *KCNMB4*, *MLLT10*, *FRG1* and *IGBP1* were visually selected. The SingleR package was utilized to identify the predominant cell types. Considering PFO closure is a process of endothelial-to-mesenchymal transition mediated fibrosis (Elliott et al., 2014), pseudotime analysis was performed on mesenchymal stem cells using to predict possible differentiation trajectory using the Monocle 2 package. Genes of interest were modeled as smooth, nonlinear function of pseudotime, and gene expression changes along this pseudotime were tested.

### 2.6 Statical analysis

Student’s t test and χ^2^ test were used to compare continuous outcomes and categorical outcomes between cases and controls in both discovery and validation cohorts. All tests were two-tailed and *p* < 0.05 was considered significant. Continuous data are presented as mean ± standard deviation (SD) and categorical data as numbers and percentages (%). All statistical analyses were conducted using R version 4.1.0 unless otherwise specified.

PLINK was used to perform genotypic association analyses. For binary traits (without PFO vs. with PFO), logistic regression was used with adjustment for the top three principal components. A *p* value threshold of less than 1 × 10^−6^ in the discovery cohort was used for suggestive genome-wide association (Schaid et al., 2018) to select candidate SNPs for validation follow-up. Quantile–quantile (QQ) plots were generated to evaluate the extent to which the observed GWAS *p* values deviated from the null hypothesis. To estimate heritability attributable to genome-wide assayed genetic variation, the genome-wide complex trait analysis (GCTA) package was used (Yang et al., 2011). A linear regression model for the size of PFO was further assessed with PLINK in the discovery cohort. The GTEx eQTL Calculator was used for expression quantitative trait loci analysis.

## 3 Results

### 3.1 GWAS of PFO in discovery cohort

**FIGURE 1:**
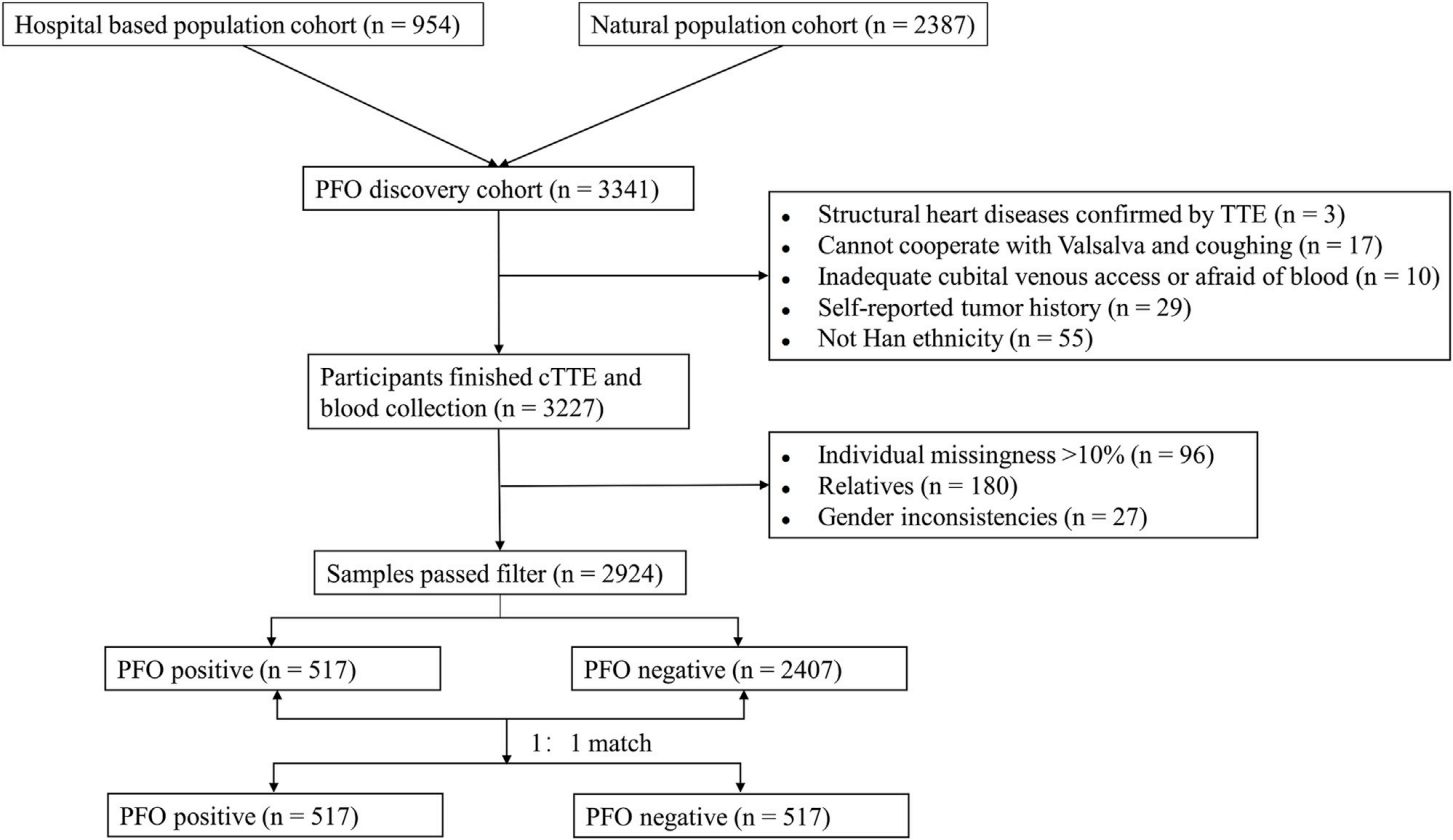
Flow chart of for screened and enrolled participants in discovery cohort.

**FIGURE 2:**
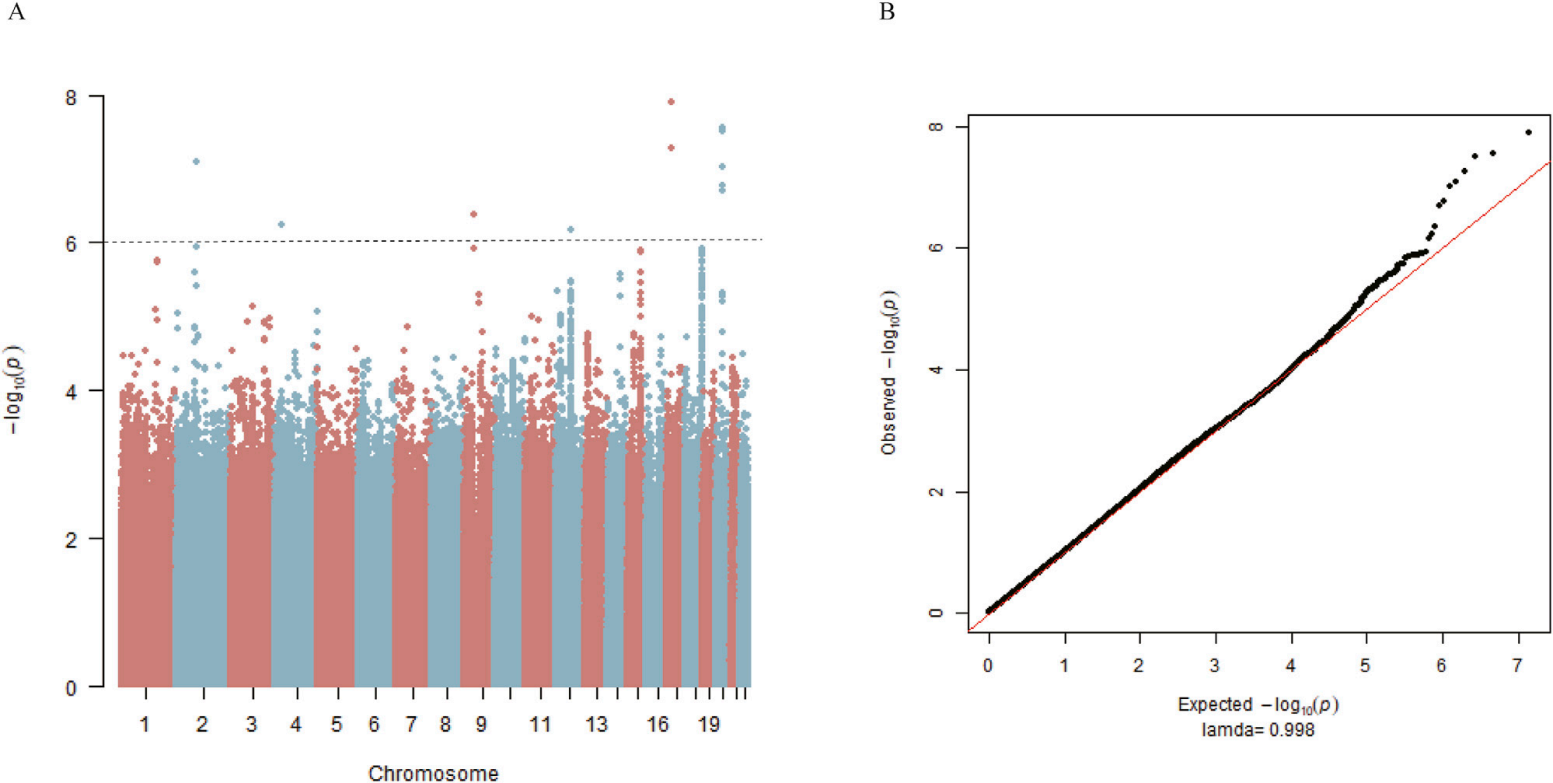
Genome-wide association study for PFO in matched 517 cases and 517 controls from the discovery cohort. **(A)** Manhattan plot summarizing the results of the association between SNPs and PFO in discovery cohort. SNPs are plotted on the *x*-axis according to their position on each chromosome in alternating colors of red and blue against the significance of the association (shown as −log10 p values) on the *y*-axis. The dashed line indicates the suggestive genome-wide significance threshold of *p* = 1 × 10^−6^. **(B)** Observed log p-values (black dots) and expected log p-values (line) are plotted versus the expected log pvalue (*x*-axis).

The study flow from initial participant screening through recruitment and selection is summarized in Figure 1. After matching demographic covariates, the discovery cohort finally included 517 PFO cases and 517 demographically matched controls (Table 1). PCA showed no stratification among participants (Supplementary Figure 1). The Manhattan plot for the main GWAS analysis is presented in Figure 2A, and the dash line marks the genome-wide suggestive significance threshold (*p* < 1 × 10^−6^). There were 3 SNPs (rs78526049, rs1313755017, rs1227675732) reaching genome-wide significance (*p* < 5 × 10^−8^) (Table 2). Among 11 SNPs reaching suggestive significance, eight were intergenic variants (rs1313755017 and rs1227675732, rs79366272 and rs78526049, rs62206791 and rs62206790 were close with a distance not more than 200bp), and three were intron variants (Table 2). The QQ plot (Figure 2B) shows the distribution of observed and expected p values, with minimal genomic control inflation (λ = 0.998). Basic heritability estimates are presented in Supplementary Table 1. After GWAS, all identified variants were confirmed through manual inspection of bam files in Integrative Genomics Viewer (IGV). Following this verification and the removal of false positives (rs62142007), Sanger sequencing was performed (Supplementary Figure 2). It was confirmed that rs1227675732, rs62206790, rs879176184, rs13115019 and rs57922961 were true positives. According to the results of GWAS in discovery cohort, *DUX4L35* (rs1227675732) (OR = 2.903, 95% CI 1.961–4.297, *p* = 3.05 × 10^−8^) showed the most significant among all true positive SNPs. A second independent association was observed at rs62206790 (OR = 2.780, 95% CI 1.864–4.146, *p* = 2.02 × 10^−7^) in *MLLT10P1*. SNPs in *FRG1HP* (rs879176184) (OR = 2.724, 95% CI 1.822–4.073, *p* = 4.30 × 10^−7^) and *IGBP1P5* (rs13115019) (OR = 2.437, 95% CI 1.702–3.488, *p* = 5.80 × 10^−7^) were also increased risk for PFO. Conversely, the rs57922961 (T > C) variant showed association with a reduced risk of PFO (OR = 0.508, 95% CI 0.388–0.666, *p* = 6.82 × 10^−7^) (Table 2).

**TABLE 2:** The SNPs associated with PFO based on case-control analysis in discovery cohort.

SNP	Position	Nearest gene	Minor allele name	Variant effect	MAF (case)	MAF (control)	p	Odds ratio (95% CI)
rs78526049	chr17: 27009297	*PDLIM1P3*	A	Intergenic variant	0.08264	0.02459	1.29E-08	3.574 (2.244, 5.690)
rs79366272	chr17: 27009277	*PDLIM1P3*	A	Intergenic variant	0.07847	0.02439	5.39E-08	3.406 (2.137, 5.430)
rs1313755017	chr20: 29459784	*DUX4L35*	A	Intergenic variant	0.09515	0.03488	2.85E-08	2.909 (1.965, 4.307)
rs1227675732	chr20: 29459788	*DUX4L35*	G	Intergenic variant	0.09496	0.03488	3.05E-08	2.903 (1.961, 4.297)
rs62142007	chr2: 90394315	*AC233266.1*	A	Intergenic variant	0.1565	0.0766	7.91E-08	2.236 (1.657, 3.016)
rs1211838747	chr20: 29087587	*FRG1DP*	A	Intron variant	0.09192	0.03393	9.51E-08	2.882 (1.924, 4.318)
rs62206791	chr20: 30418210	*MLLT10P1*	C	Intergenic variant	0.09792	0.03774	1.68E-07	2.768 (1.864, 4.110)
rs62206790	chr20: 30418174	*MLLT10P1*	A	Intergenic variant	0.09708	0.03723	2.02E-07	2.780 (1.864, 4.146)
rs879176184	chr9: 41008177	*FRG1HP*	C	Intron variant	0.09129	0.03557	4.30E-07	2.724 (1.822, 4.073)
rs13115019	chr4: 28719069	*IGBP1P5*	A	Intergenic variant	0.1036	0.04528	5.80E-07	2.437 (1.702, 3.488)
rs57922961	chr12: 70345617	*CNOT2*	C	Intron variant	0.08915	0.1615	6.82E-07	0.508 (0.388, 0.666)

### 3.2 GWAS for PFO grade

Among the 517 subjects with PFO, 121, 125 and 271 cases were categorized as Grade 1, 2 and 3 respectively. We observed that mutations in *DUX4L35* (rs1227675732), *MLLT10P1* (rs62206790), *FRG1HP* (rs879176184), *IGBP1P5* (rs13115019) mainly presented in PFOs with large shunt (Grade 3) (Supplementary Figure 3A). The SNP rs57922961, associated with a reduced risk of PFO, was mainly found in PFOs with moderate-to-large shunts (Grade 2–3) (Supplementary Figure 3A). Exploratory analyses of quantitative measures of PFO were also conducted. None of the SNPs analyzed met conventional criteria for genome-wide significance (Supplementary Figure 3B). However, rs62206790, rs1227675732, rs13115019 and rs879176184 showed nominal evidence of association in PFO size (*p* < 0.01) (Supplementary Table 2). The QQ plot illustrated observed p values were smaller than those expected, likely due to insufficient sample size for detecting effects on PFO size (Supplementary Figure 3C).

### 3.3 Identification of SNPs in validation cohort

**FIGURE 3:**
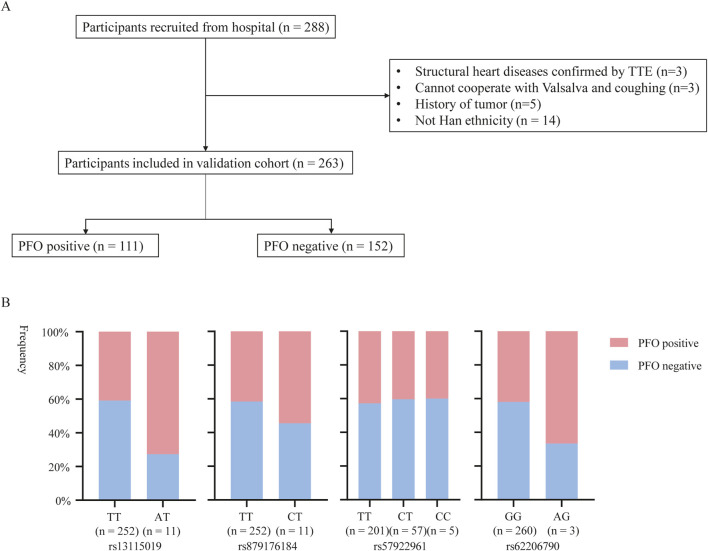
The potential SNPs associated with PFO entered in the validation stage. **(A)** Flow diagram of included participants in validation cohort. **(B)** rs13115019, rs879176184, rs57922961 and rs62206790 mutations of PFO in validation cohort.

Replication genotyping was performed in a validation cohort of 111 PFO cases and 152 controls (Table 3). The flow chart of patient enrollment of validation cohort is shown in Figure 3A. There were 66.7% cases (74/111) with large shunt. The variations of rs62206790, rs879176184, rs13115019 were more prevalent in participants with PFO, and the variation of rs57922961 was less common in participants with PFO (Figure 3B). The trends of these four SNPs variation in PFO were recapitulated in the validation cohort, although the differences were not significant at the nominal level (*p* = 0.0576, *p* = 0.3972, *p* = 0.7383, *p* = 0.3883, respectively). However, individuals with ≥2 of these four site variations were much more likely to have PFO in the validation cohort (*p* = 0.0109). The rs1227675732 variation was not observed in the validation cohort.

**TABLE 3:** Characteristics of participants with or without PFO in validation cohort.

	Without PFO (n = 152)	With PFO (n = 111)	*p*
Age, mean (SD)	35.35 (15.08)	31.06 (12.04)	**0.014** ^b^
Gender, female (%)	99 (65.1)	71 (64.0)	0.948^a^
BMI, mean (SD)	22.46 (3.24)	22.21 (3.56)	0.549^b^
Smoker, n (%)	17 (11.2)	14 (12.6)	0.872^a^
Alcohol drinker, n (%)	18 (11.8)	16 (14.4)	0.669^a^
Coffee drinker, n (%)	18 (11.8)	16 (14.4)	0.669^a^
Education, n (%) <6 years 6–9 years 10–12 years >12 years	5 (3.3) 13 (8.6) 30 (19.7) 104 (68.4)	3 (2.7) 8 (7.2) 27 (24.3) 73 (65.8)	0.823^a^

Bold indicates significant differences at p < 0.05.

^a^ χ2 test.

^b^ Student’s t test.

### 3.4 Characterization of GWAS loci

**FIGURE 4:**
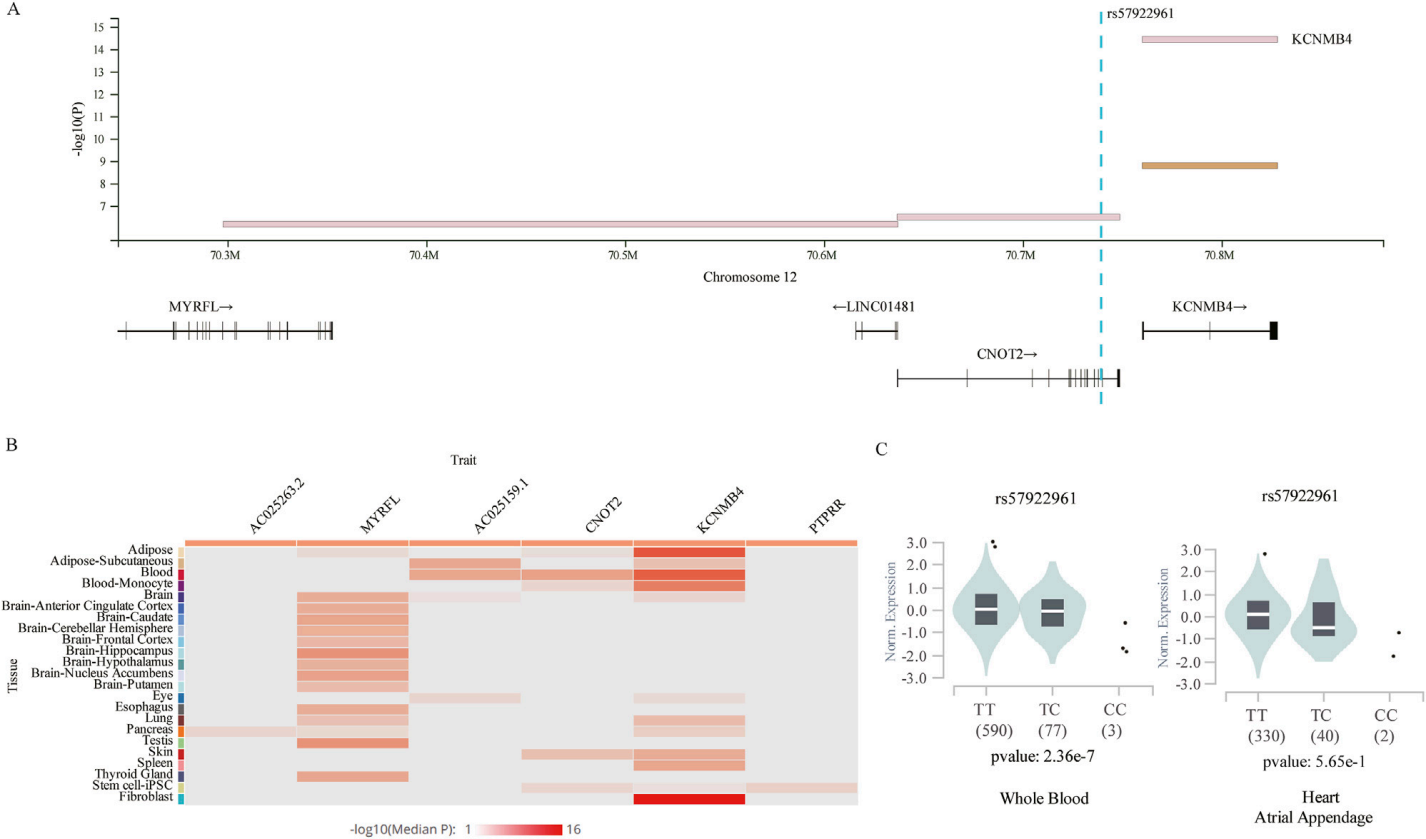
The expression quantitative trait loci (eQTLs) analysis for rs57922961. **(A)** The chromosomal location of rs57922961 based on GTEx Project. **(B)** Heatmap showing average gene expression values related to rs57922961 in different human tissues from the GTEx portal. **(C)** The correlation of rs57922961 genotypes and corresponding mRNA expression levels in whole blood cells and normal heart atrium tissue.

Given that the four identified SNPs were in intergenic or intronic regions, their functional potential was annotated using publicly available databases. We focused on rs57922961 and its associated gene expression (Figure 4A), while *MLLT10P1* (rs62206790), *FRG1HP* (rs879176184) and *IGBP1P5* (rs13115019) could not be tested for eQTLs in whole blood or heart tissues according to GTEx Project datasets. This SNP was found to modify expression levels of *KCNMB4*, a gene 11.3 kb downstream of *CNOT2*, based on the GTEx database (Figure 4A). Figure 4B showed the expression levels of genes correlated with rs57922961 in different tissues. We found that the rs57922961-T allele was associated with higher expression levels in whole blood (*p* = 2.36 × 10^−7^) but was not significantly correlated with mRNA expression levels in heart atrium tissue (*p* = 0.565) (Figure 4C).

**FIGURE 5:**
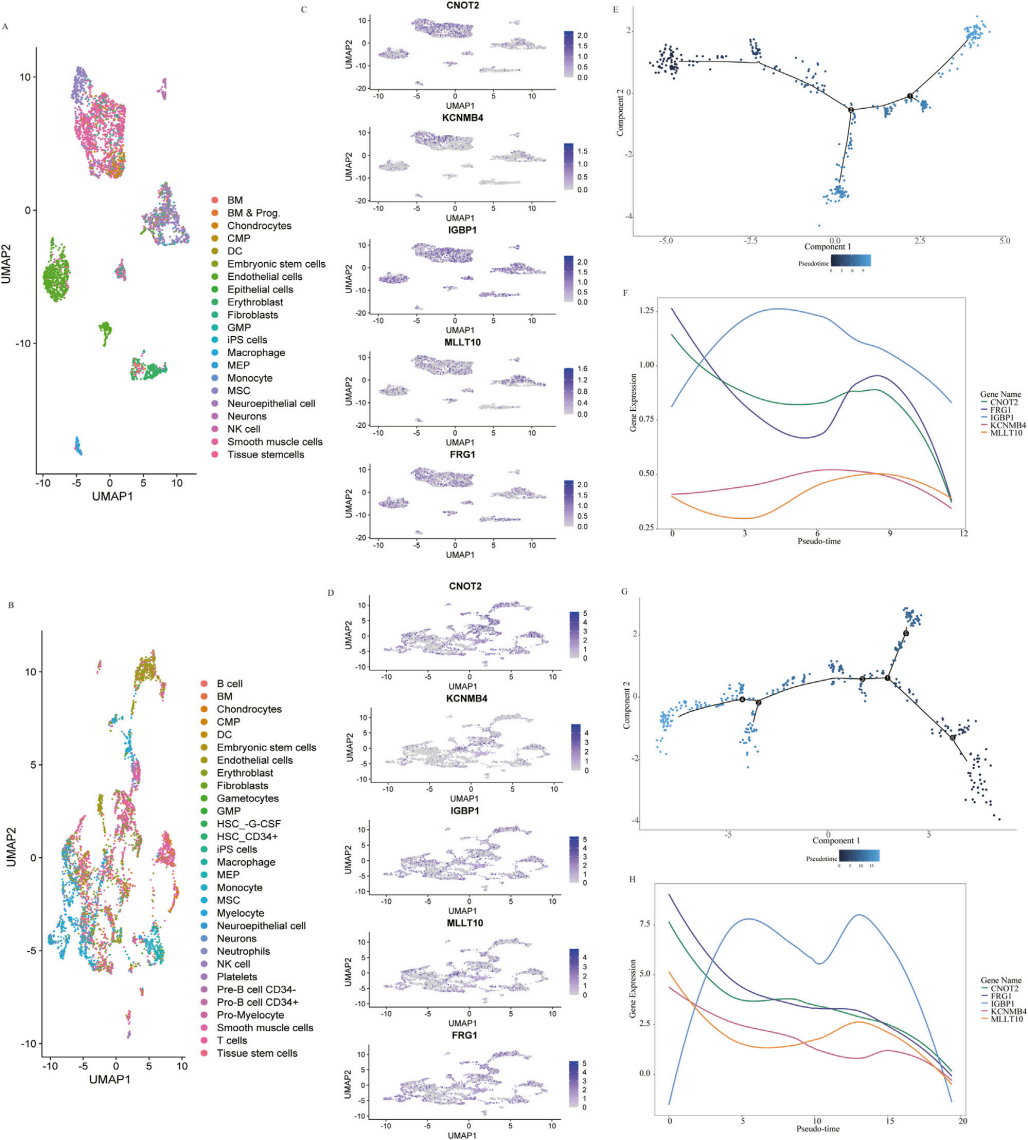
Single-Cell Analysis of 4.5–9 gestational weeks (W) (data from Asp et al., 2019) and 5–25 W (data from Cui et al., 2019) human embryonic hearts. Dimensionality reduction from the 4.5–9 W **(A)** and 5–25 W **(B)** hearts colored by cluster affiliation. The identified cell types, which are annotated based on marker gene information, are indicated on the right. The detection of CNOT2, KCNMB4, IGBP1, FRG1 and MLLT10 expression across scRNA-seq of 4.5–9 W **(C)** and 5–25 W **(D)** heart. Pseudotime trajectory showing the distribution of cardiac mesenchymal stem cells **(E)** and curves showing dynamic expression changes in five genes along pseudotime trajectory **(F)** in 4.5–9 W hearts. Pseudotime trajectory showing the distribution of cardiac mesenchymal stem cells **(G)** and curves showing dynamic expression changes in five genes based on pseudotime **(H)** in 5–25 W hearts.

To further explore the function of the genes in heart development, we leveraged publicly available human single-cell RNA-seq data of heart tissue. The single-cell RNA sequencing data from Asp et al. (2019) were generated from 19 heart tissue sections from the 4.5 to 9 post-conception weeks, and the data from Cui et al. (2019) were generated from 18 human embryos’ hearts ranging from 5 to 25 weeks of gestation. Dimensionality reduction and cell type categorization were performed based on marker gene expression for the filtered cells in the two datasets separately (Figures 5A, B). As *FRG1HP*, *MLLT10P1* and *IGBP1P5* are pseudogenes, we replaced them with their homologous functional genes. Figures 5C, D revealed the expression of *FRG1*, *MLLT10*, *IGBP1*, *CNOT2* and *KCNMB4* in distinct populations of cells in 4.5–9-week and 5–25-week hearts, separately. Monocle2 pseudotime trajectory analyses were used to develop a developmental course of mesenchymal stem cells (MSC). Two branch points were identified in 4.5–9-week hearts (Figure 5E) with *KCNMB4* and *IGBP1* expression declining after an initial increase (Figure 5F). *CNOT2*, *MLLT10* and *FRG1* showed a drop in expression with a slight increase in the middle across pseudotime in 4.5–9-week hearts (Figure 5F). The same pseudotime analysis in the 5–25-week hearts also identified cell fates in an unsupervised manner (Figure 5G). It also identified heightened expression of *IGBP1* at the beginning, with decreased expression along the pseudotime trajectory (Figure 5H). Restricting the analyses to hearts in the 5–25 W stage, similar results were observed with *CNOT2*, *MLLT10*, *FRG1* and *KCNMB4* showing slight increases during the descent process along pseudotime.

## 4 Discussion

Using a pragmatic approach of PFO diagnosis based on routine clinical practice and international recommendations, we performed a GWAS for PFO. We detected rs62206790, rs879176184, rs13115019 and rs57922961 as suspect loci. To our knowledge, this is the first GWAS focused on genetic contributions in PFO.

Previous clinical study illustrated that PFO was a familial trait with a genetic basis (Arquizan et al., 2001). We reviewed publicly available studies evaluating genetic anomalies of PFO. It had been reported that *NOTCH3* involved in cardiac development, was related to cerebral autosomal dominant arteriopathy with subcortical infarcts and leukoencephalopathy (CADASIL) that might share genetic pathways with PFO (Dulamea et al., 2019). Animal experiments also supported Notch signaling was an important player participating in the physiological process of foramen ovale closure (Elliott et al., 2014). Other potential genes involved in the development of cardiovascular system (*GATA4*, *TBX20*, *NKX2-5*) were also recognized to be pathogenic for PFO (Kirk et al., 2006; Paolucci et al., 2021). A recent study on the whole-exome sequencing on 25 patients with PFO also suggested *NKX2-5* might play a role in PFO development (Li et al., 2024). Nevertheless, further studies confirmed these variants were likely to be relatively benign in terms of genetic participation in PFO, or were still lack of significant association in population-based data (Belvis et al., 2009; Daghals et al., 2022; Moradi et al., 2011). Additionally, results from human and animal studies illustrated that the mutation of *NKX2-5* was not common and usually cause severe congenital heart diseases, including atrial septal defect, double-outlet right ventricle, or Fallot, along with PFO (Biben et al., 2000; Du M et al., 2016; Elliott et al., 2003; 2010). Large genetic studies of PFO are still lacking to date, which might be limited by the complex screening methods. It is commonly believed that transesophageal echocardiography (TEE) is the best method for detecting a PFO; however, it is difficult to achieve in population screening for its more invasiveness and distress (Schneider et al., 1996). Transcranial Doppler (TCD) is another method of detecting PFO with high sensitivity, while it has a limited ability to differentiate intracardiac from extracardiac shunts, such as pulmonary arteriovenous malformation, arteriovenous fistula thrombus, and parietal arteriovenous malformation (Chen et al., 2023; Liu et al., 2021; Mojadidi et al., 2015). In addition, if the shunt is due to other congenital heart diseases, like atrial septal defect or ventricular septal defect, TCD will not be able to distinguish these entities from a PFO(Homma et al., 2016). Another technique for PFO screening is cTTE, which has been proven to be more specific compared to TCD (Katsanos et al., 2016; Van der Giessen et al., 2020). In the early 1990s, the sensitivity of cTTE was about 60%, but it has increased to 80%–90% in recent studies, probably as a result of significant advancements in ultrasound imaging (Daniels et al., 2004; Lanzone et al., 2024; Ren et al., 2013; Takaya et al., 2022). We also measured pressure during the performance of the Valsalva maneuver, which could increase the sensitivity of detecting PFO to more than 90% (Takaya et al., 2020; Zhao et al., 2019). Nowadays, many large-scale studies of PFO, like MIST, NOMAS, RESPECT trials, enroll patients on the basis of cTTE (Ben-Assa et al., 2020; Dowson et al., 2008; Mas et al., 2001; Rundek et al., 2008; Saver et al., 2017). Additionally, cTTE with the combination of TTE could also help to exclude the bias of congenital heart diseases with PFO (Kheiwa et al., 2020). Thus, we screened PFO by cTTE with comparative sensitivity and specificity in this large-scale genome wide association study to define genetic susceptibility to PFO without other congenital heart diseases.

In this study, we found an association between rs57922961 and foramen ovale closure. *CNOT2*, closest to rs57922961, is a member of the CCR4-NOT complex which is a master regulator of multiple cellular processes (Niceta et al., 2023). The major repression function of *CNOT2* is localized in a specialized protein motif, the Not-Box, which is sensitive to the histone deacetylase inhibitor (Zwartjes et al., 2004). The high expression of histone deacetylase is involved in different phenotypes of heart disease, including myocardial hypertrophy, myocardial fibrosis, ventricular dilatation, arrhythmia, cardiac dysfunction, etc. (Jin et al., 2023) It has been reported that silencing *CNOT2* could affected cardiac chamber size and contractility, which also proved that CCR4-NOT complex played crucial role in heart development and function (Elmen et al., 2020). Meanwhile, *CNOT2* was identified as the critical gene for in the development of human embryonic cells (Zukeran et al., 2016). *CNOT2* was downregulated during the neural development (Chen et al., 2011), which is similar to our findings in the heart development. Furthermore, rs57922961 is also related to the expression of *KCNMB4* forming a subunit of potassium calcium-activated channel which are fundamental to the control of smooth muscle tone and neuronal excitability (Bertrand et al., 2020; Feng et al., 2020). Although developed mammalian cardiac cells do not exhibit functional expression of potassium calcium-activated channel, evidence suggests its presence in cardiac fibroblasts (Chen et al., 2024). In human adults, fibrotic tissues can be observed around the septum primum which is formed by a cap of mesenchymal tissue derived from the endocardial cushion playing an important role in the closure of foramen ovale (Hara et al., 2005).

Our results also suggested that *MLLT10P1* (rs62206790), *FRG1HP* (rs879176184), *IGBP1P5* (rs13115019) were associated with the risk of PFO. Human pseudogenes are usually disabled gene homologues, however, approximately 19% of pseudogenes could be transcribed playing key evolutionary and regulatory roles (Qian et al., 2022). Pseudogenes can compete with their parent genes for microRNA (miRNA) binding, modulating the repression of the functional gene by its cognate miRNA (Pei et al., 2012). It has been reported that several pseudogenes are related to cardiac diseases via gene conversion, homologous recombination, as antisense RNA or endogenous small interference RNA to regulate the expression of their parental genes (Wu et al., 2020). In our study, all parent genes of *MLLT10P1*, *FRG1HP*, *IGBP1P5* expressed in the development of heart and showed decreased expression trends along the pseudotime trajectory, suggesting these SNPs might play a role in heart development. The Human Phenotype Ontology (HBO) project shows *IGBP1*, the parent gene of *IGBP1P5*, is related to ventricular septal defect and patent ductus arteriosus (Gargano et al., 2024). *IGBP1* is expressed as a 1.4-kb mRNA in the peripheral blood leukocytes, fetal liver, heart, brain, pancreas, placenta, etc., (Onda et al., 1997) It is involved in regulation of the catalytic activity of the phosphatases PP2A and the IGBP1-PP2Ac dimer could bind to PI3K, which furtherly involved in the regulation of PI3K/AKT pathway (Nadel et al., 2022). HBO also shows the parent gene of *FRG1HP*, *FRG1*, is associated with abnormal cardiovascular system morphology (Gargano et al., 2024). The altered Frg1 levels could lead to abnormal process of endothelial-to-mesenchymal transition (Hansda et al., 2017), which is an important step during the closure of foramen ovale. As for *MLLT10*, it had been reported that its suppression resulted in downregulation of fibroblast-specific genes and accelerated the activation of pluripotency-associated genes, which played a role in development (Ugurlu-Cimen et al., 2021). Besides, the expression of *MLLT10* was associated with pain perception/maintenance, including migraine (Rahmioglu et al., 2023), the occurrence of which was closely related to PFO. However, functional verification of these SNPs and their parent genes should be furtherly studied. The homologous functional gene of DUX4L35, DUX4, is associated with several diseases, including skeletal muscle atrophy and human end-stage cardiomyopathy (Mocciaro et al., 2021), while it is epigenetically silenced in most somatic tissues of healthy humans (Movassagh et al., 2011), and its expression could not be detected in the single-cell gene expression datasets. Thus, combined with the results of our validation cohort, the rs1227675732 needs to be interpreted with caution.

While the study provides risk loci for PFO, several limitations should be acknowledged. First, the same tendency was observed but the SNPs did not reach significance in the validation cohort, and larger cohorts are needed in further study. Second, our analyses did not consider other forms of genetic or epigenetic variation. The variants segregating within families are also needed to be indicated in the future. Third, our analyses were performed in Chinese individuals with PFO, and whether our findings are generalizable to other populations should be further studied. Fourth, direct cell or animal experiments of how the identified SNPs influence PFO development need to be explored in the future. Fifth, though cTTE had its strengths and we tried to overcome its known limits, the results should be interpreted with care due to the intrinsic limitations of the technique. Besides, variables not included as covariates in the PSM analysis may still lead to potential bias. Despite the limitations present in our study, our findings lay a pivotal groundwork for future exploration into the inheritance of PFO. GWAS study investigating potential common variants in PFO could pave the way for pointing out PFO genetic architecture. Moreover, we analyzed several risk loci of PFO and their expression in different tissues, which provide potential biological mechanisms of heart development.

## 5 Conclusion

Our study may offer fresh perspectives on the involvement of SNPs in the pathogenesis of PFO and establish a foundation for forthcoming investigation aimed at interventional indication of PFO and new therapeutic targets for PFO-related diseases.

## Data Availability

The original contributions presented in the study are publicly available. This data can be found here: https://github.com/betsy1226/PFO.
