# Supplementary material for: Genetic variation in patent foramen ovale: a case-control genome-wide association study
Source: Front Genet. 2025 Jan 13;15:1523304. doi: 10.3389/fgene.2024.1523304 (PMC11769951; doi:10.3389/fgene.2024.1523304)
Supplement: Supplementary file 1 [file DataSheet1.docx]

Supplementary Material

# Supplementary Data

Primers used for confirmation of mutations with Sanger:

PDLIM1P3-F GAAGCACAAACACCACGGAC

PDLIM1P3-R AGATACGCCCAGACACACAG

DUX4L35-F TGTGTCTGTGTGTCCTTAGTGT

DUX4L35-R AGGAATTTAGGCTTGGACTGGA

FRG1DP-F AGACCTGCCGAGAACTTTTG

FRG1DP-R TCTGCCTTCATTATGTGCTTGG

MLLT10P1-F CCATCTCGGCCTTCCAAGTA

MLLT10P1-R ACAGAGCAGGGTCAAAAGGG

FRG1HP-F GCAGAGGCGGGTGAATCA

FRG1HP-R ATAGCAACATACAGCCAGGC

IGBP1P5-F AGCCCTACCTGTGATAGATACA

IGBP1P5-R CACCCGAGATCAGGAATTCG

CNOT2-F TCACTGGCAGTTCTGTTTTCAT

CNOT2-R ACTTCACTCCTTTCTTGCTCTTC

# Supplementary Figures and Tables

## Supplementary Tables

**Supplementary Table 1.** **Heritability of patent foramen ovale.**

| Source | Variance | SE |
| --- | --- | --- |
| V(G) | 0.107291 | 0.090544 |
| V(e) | 0.145587 | 0.089668 |
| Vp | 0.252878 | 0.011279 |
| V(G)/Vp | 0.42428 | 0.355905 |

V(G), genotypic variance; V(e), error variance; Vp, total phenotypic variance; trait heritability, h2 = V(G)/Vp

**Supplementary Table 2.** **The SNPs associated with PFO based on quantitative measures of PFO analysis in discovery cohort.**

| SNP | Position | BETA | STAT | *p* |
| --- | --- | --- | --- | --- |
| rs1227675732 | chr20: 29459788 | 0.3214 | 3.519 | 0.0004708 |
| rs62206790 | chr20: 30418174 | 0.3363 | 3.556 | 0.0004137 |
| rs879176184 | chr9: 41008177 | 0.2753 | 2.855 | 0.004486 |
| rs13115019 | chr4: 28719069 | 0.2609 | 3.519 | 0.001821 |
| rs57922961 | chr12: 70345617 | -0.13510 | -1.5230 | 6.82E-07 |

## Supplementary Figures


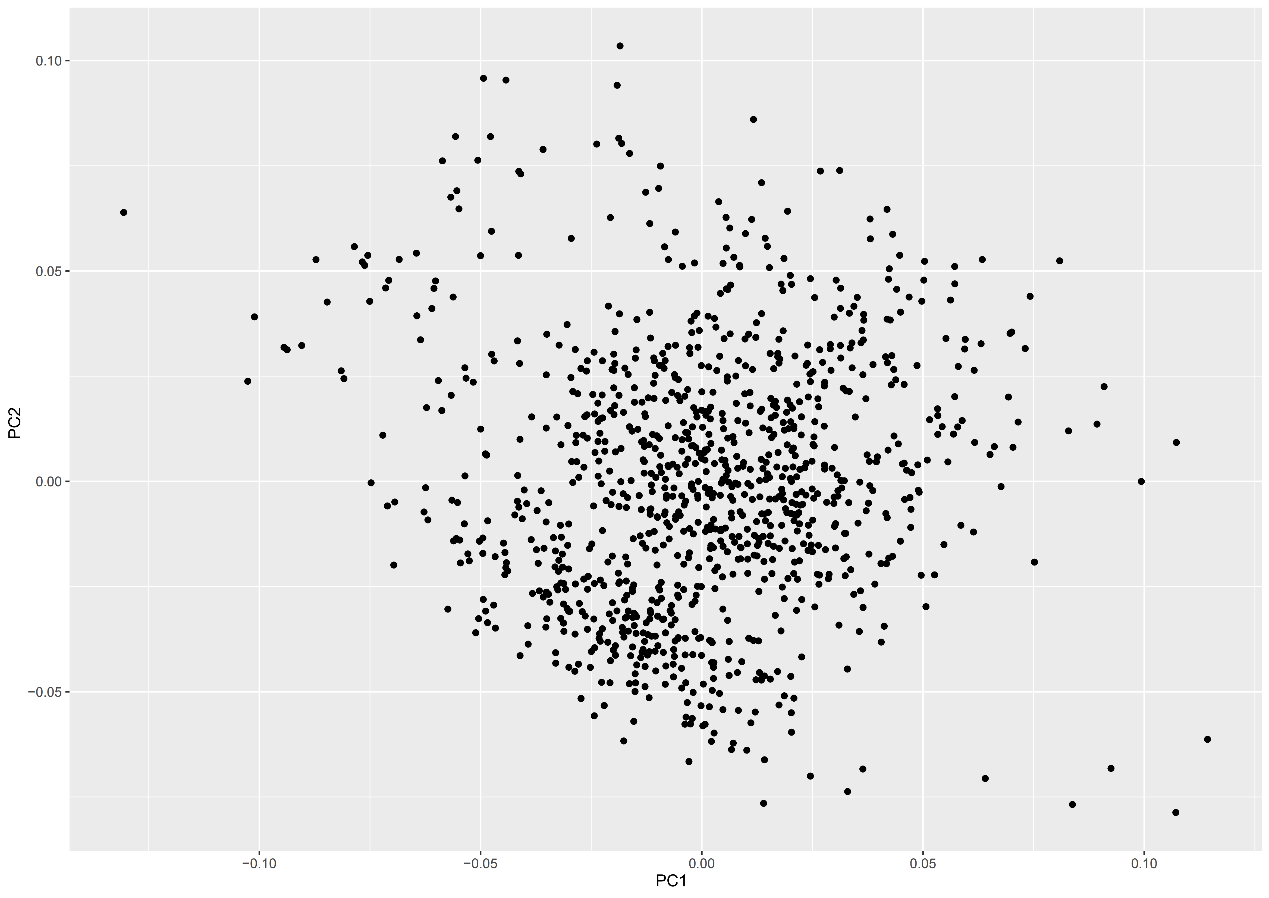


**Supplementary Figure 1.** Principal components analysis (PCA) was performed to evaluate genetic homogeneity.


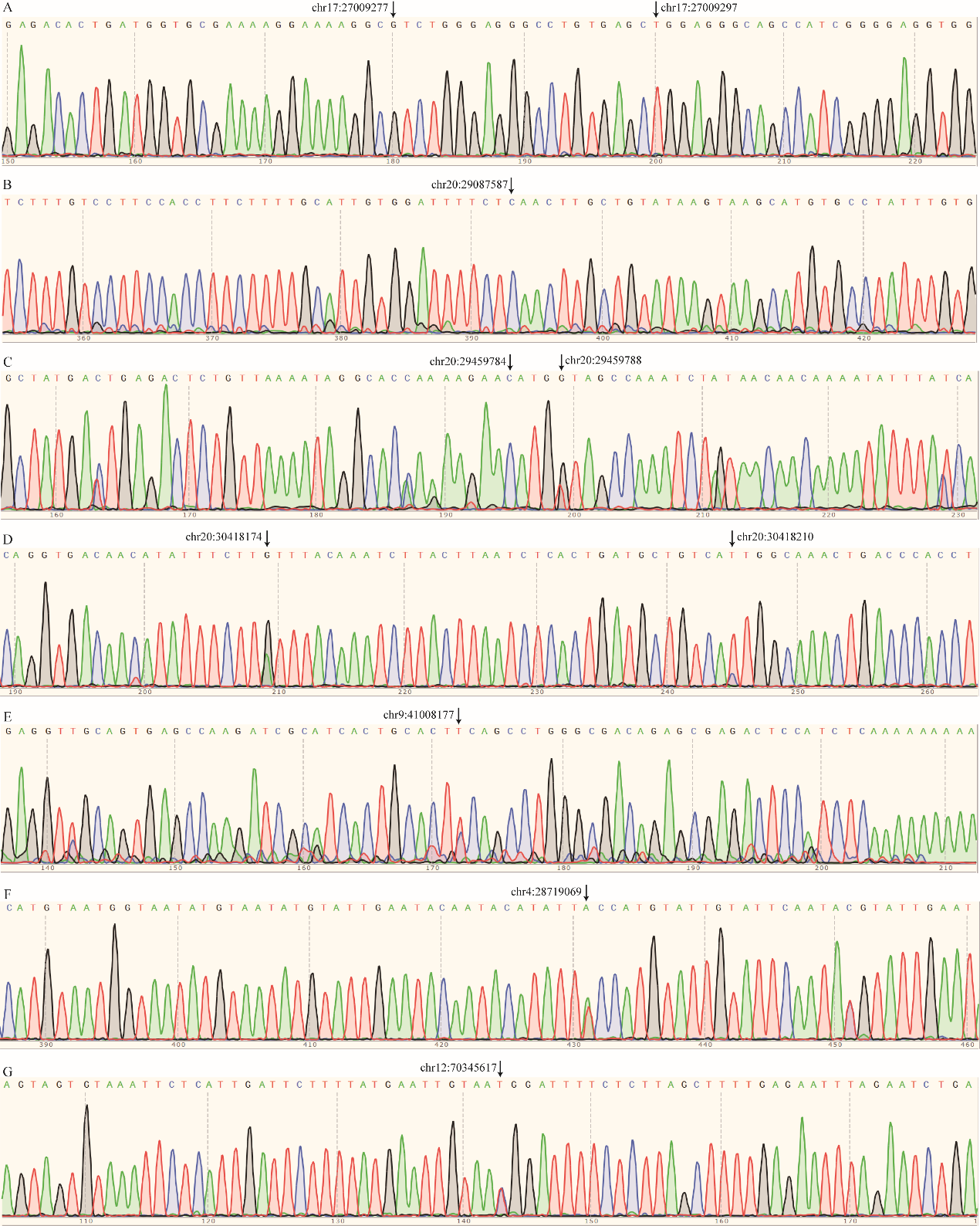


**Supplementary Figure 2.** Validation of SNPs with Sanger sequencing.


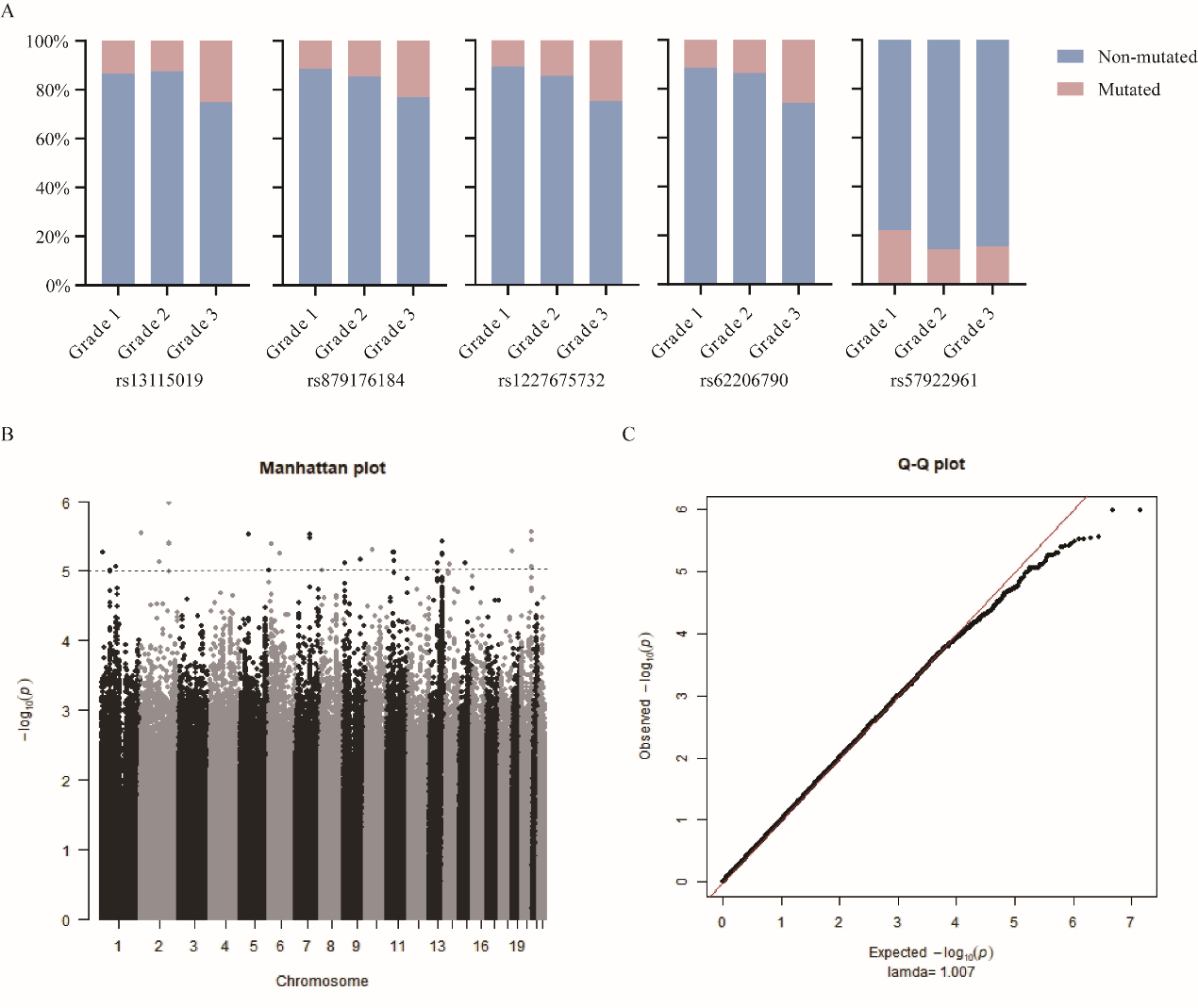


**Supplementary Figure 3.** Genome-wide association study for PFO size (121 cases for Grade 1, 125 cases for Grade 2, and 271 cases for Grade 3) from the discovery cohort. (A) rs13115019, rs879176184, rs57922961, rs1227675732 and rs62206790 mutations in different PFO Grade. (B) Manhattan plot summarizing the results of the association between SNPs and PFO size in discovery cohort. The dashed line indicates the suggestive genome-wide significance threshold of *p*=1×10^-5^. (C) Observed log p-values (black dots) and expected log p-values (line) are plotted versus the expected log pvalue (x-axis).
